# Neurological and Vascular Manifestations of Ethylmalonic Encephalopathy

**Published:** 2017

**Authors:** Ali Reza TAVASOLI, Parastoo ROSTAMI, Mahmoud Reza ASHRAFI, Parvaneh KARIMZADEH

**Affiliations:** 1 Pediatric Neurology Division, Neurometabolic Registry Center, Children’s Medical Center, Tehran University of Medical Sciences, Tehran, Iran; 2 Division of Endocrinology and Metabolism, Department of Pediatrics, Children’s Medical Center, Tehran University of Medical Sciences, Tehran, Iran.; 3 Pediatric Neurology, Pediatric Neurology Research Center, Shahid Beheshti University of Medical Sciences, Tehran, Iran; 4 Pediatric Neurology Excellence Center, Pediatric Neurology Department, Mofid Children Hospital, Faculty of Medicine, Shahid Beheshti University of Medical Sciences (SBMU), Tehran, Iran

**Keywords:** Ethylmalonic Encephalopathy, ETHE1 Gene Mutation, Neurologic Manifestations, Vascular Manifestations

## Abstract

**Objective**

Ethylmalonic encephalopathy (EE) is a severe mitochondrial disease of early infancy clinically characterized by a combination of developmental delay, progressive pyramidal signs, and vascular lesions including petechial purpura, orthostatic acrocyanosis, and chronic hemorrhagic diarrhea.

Biochemical hallmarks of the disease are persistently high level of lactate, and C4–C5-acylcarnitines in blood, markedly elevated urinary excretion of methylsuccinic and ethylmalonic (EMA) acids. Here we report two patients with EE as a 16-months-old male infant and a 2-yr-old boy referred to Pediatric Neurology Clinic in Children’s Medical Center, Tehran-Iran that in one patient genetic analysis revealed a homozygous mutation of the ETHE1 gene in favor of ethylmalonic acidemia.

## Introduction

Ethylmalonic encephalopathy (EE) (OMIM602473), is a very rare mitochondrial disorder caused by mutations in the ETHE1 gene localized on chromosome 19q13 (1). The ETHE1 protein is a 30 KD polypeptide located on the mitochondrial, Fe-containing sulfur dioxygenase (SDO) activity and involved in catabolism of sulfide. Loss of function ETHE1 caused by impaired activity of this protein leads to the accumulation of H2S (dihydrogen sulfide) and derivative thiosulphate in crucial tissues, including liver, brain and colonic mucosa. Increased concentration of these toxic products inhibits SCAD (short-chain Acyl-CoA dehydrogenase deficiency) and COX (Cytochrome c oxidase) activities; therefore, accounting for EMA aciduria and high serum levels of C4- and C5-acylcarnitines and lactate (2). 

Ethylmalonic encephalopathy is clinically characterized by the early onset of neurological degeneration, hypotonia, psychomotor retardation, seizure, spastic tetraplegia, Leigh-like syndrome, recurrent petechiae, chronic hemorrhagic diarrhea and orthostatic acrocyanosis, leading to death in the first years of life. 

The accumulation of H2S damages the endothelial cells and vasodilation, which account for the petechiae and the acrocyanosis. H2S overload in colonic mucosa result in ulcerative colitis by reduced detoxification (3, 4). 

The prognosis is poor; therefore, half of the patients die within the first 2 years of life from metabolic decompensation (5). We emphasize that physician should consider Ethylmalonic Encephalopathy in all patients with neurological deterioration, lactic acidosis combination with vascular manifestations.

Here we report two patients with EE as a 16-months-old male infant and a 2-yr-old boy.

## Case presentation

**Patient consent: **Written informed consent was obtained from parents.

**Case 1**

**Fig 1:**
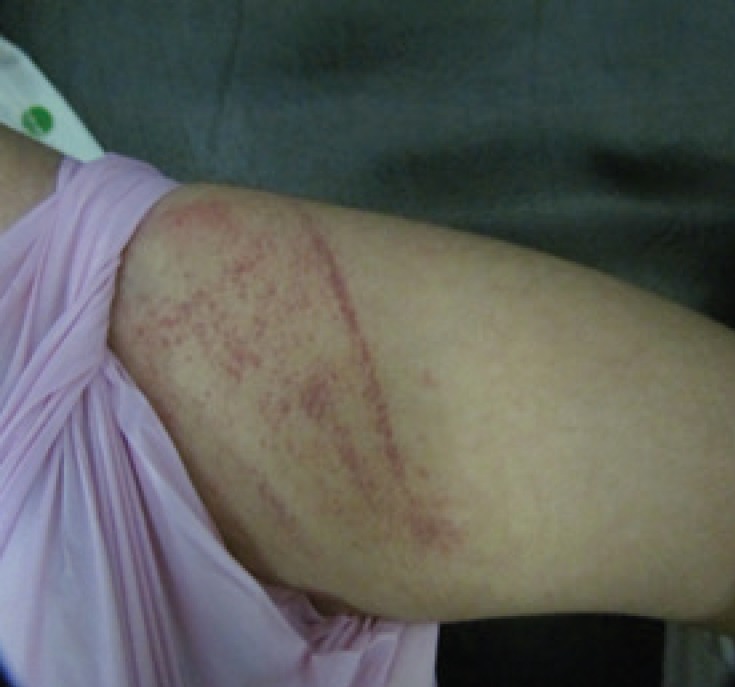
Petechiae lesions on the thigh of the patient 1

**Fig 2:**
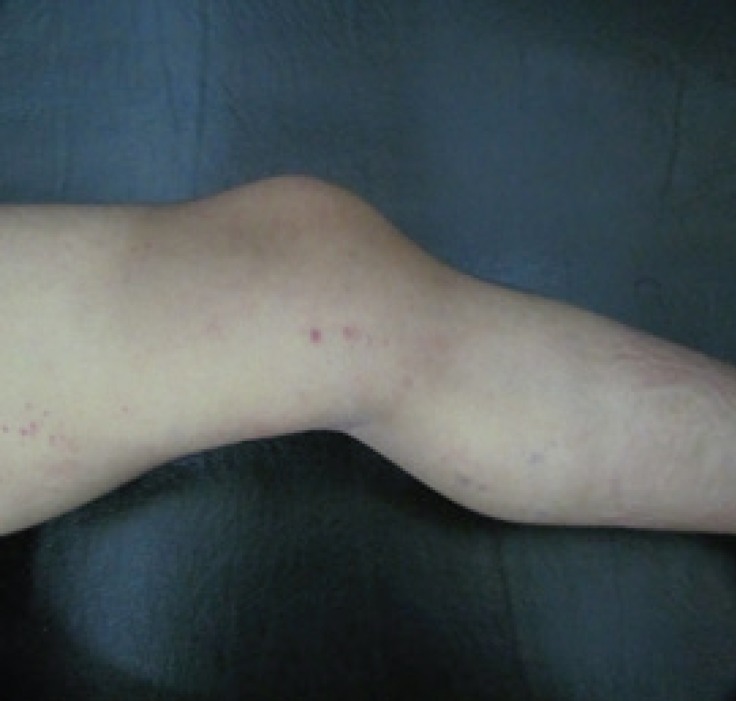
Petechiae lesions on the leg of the patient1

A 16-months-old male infant was referred to Pediatric Neurology Clinic in Children’s Medical Center, Tehran-Iran because of seizure, developmental delay, and petechiae in 2016. He was the second child of consanguineous parents, born with a weight of 2500 gr, length of 44cm, and a head circumference of 36 cm. The first child had dead at the age of three years following frequent severe convulsions without making a diagnosis. On neurologic examination, he had global developmental delay and axial hypotonia, exaggerated deep tendon reflexes, inability to sitting and standing but he was able to keep the head. In addition, he had diffuse petechiae on his examination (Figure 1, 2).

Laboratory tests revealed an elevation of leucine (315μmol/L, norm30-246) and isoleucine (200μmol/L, norm 6-122) in serum amino acid analysis. Lactic acid levels had been elevated in plasma (5 MM; normal range: 0.7–1.8) and in CSF (7.4 MM; normal range: <2.2) with increased lactate/pyruvate ratio (19 and 24 in plasma and CSF, respectively). Serum ammonia and acylcarnitine profile, liver function test were normal.

Urine organic acid showed high ethylmalonic acid (1200 MM/M creatinine, norm<17). MRI (Magnetic Resonance Imaging) showed abnormal signal in basal ganglia especially caudate and dentate nucleus and some degree of atrophy in corpus callosum. To confirm the diagnosis of EE, mutation analysis was performed that revealed a homozygote ETHE1 gene mutation compatible with diagnosis of ethylmalonic acidemia.

Currently, the patient is 2 yr old; he has developmental delay, axial hypotonia, hypertonia, and hyperreflexia in limbs. He has also episodes of generalized seizures despite antiepileptic treatment. At the time being, he is mainly treated with mitochondrial cocktail. 

**Case 2**

A 2-year-old boy was referred to Pediatric Neurology department of Mofid Children Hospital, Tehran-Iran for evaluation of neurodevelopmental delay. He was the first child of a consanguineous marriage, born with a weight of 3100 gr, length of 50 cm, and a head circumference of 36 cm. He had rolling and creeping but did not have the ability to sit, standing and walking. He could not speak. 

He had a history of admission for bloody stool after 20 days of birth but hematologic and intestinal evaluations did not confirm abnormal evidence. Neurological exam showed cerebral hypotonia. MRI revealed abnormal signal intensity in periventricular white matter and basal ganglia. Laboratory exams showed no specific change but serum lactic acid was mildly elevated (3 MM; normal range: 0.7–1.8). According to clinical manifestations and suspicion to neurometabolic disorders, metabolic evaluation included urine organic acid was requested that showed significant elevated ethylmalonic acid (2125 MM/M creatinine, normal<17). Genetic study to confirm the diagnosis of EE was not done in this patient. 

## Discussion

An increasing number of patients with EE are being diagnosed. The actual incidence of the condition may be significantly underestimated because the biochemical phenotype and clinical manifestations may be misinterpreted to other metabolic disorders (6). 

Overall, 145 patients with different clinical manifestations have been reported since 1993 (3-20). The cardinal symptoms of EE are neurologic and vascular but congenital anomalies of the central nervous system (tethered cord, Chiari I malformation), moderate hydronephrosis and undescended testes, cardiac involvement, respiratory failure, nephrotic syndrome, crescentic glomerulonephritis, articular hyperlaxity, Neonatal Marfan phenotype, west syndrome and dysmorphic feature have been described (3-20). Most of patients have a severe form of the disorder with infantile onset and die within the early years of life. A few patients have a chronic form of the disease (6). 

Our patients showed neurologic and vascular symptoms include hypotonia, developmental delay, refractory seizure, petechiae, and bloody stool. Case 2 was managed mainly for hematologic and gastrointestinal disease regardless of neurologic symptoms. The diagnosis of EE was based on combination of clinical presentations, lactic acidosis, remarkable elevated urinary level of ethylmalonic acid and C4-6 acylglycines, high serum levels of C4- and C5-acylcarnitines and methylsuccinic acid (3). As a metabolic disease was suspected from early stages of disease in our cases, mutation analysis was done in case 1 following a standard protocol including PCR amplification and Sanger sequencing of coding exons and adjacent intron of the ETHE1 gene. 

Hereby, the mutation c.C487T, in a homozygous state, was found in exon 4. Both parents were heterozygous for this mutation, confirmed obligate parental carrier status and segregation of the mutation. After first report of this mutation (1), about 31 ETHE1 gene mutations were reported (the Human Gene Mutation Database). In the most of cases, mutations lead to absence of ETHE1 protein due to partial gene deletion, degradation of premature termination codon-containing ETHE1 transcripts by nonsense-mediated decay, or by degradation of misfolded ETHE1 proteins by quality control proteases (6).

**In conclusion, **if skin petechia or other hemorrhagic manifestations such as bloody stool are seen in association with other evidence of organic academia in biochemical and imaging evaluations in a patient under investigation for neurometabolic disorders; ethylmalonic academia should be considered in mind.
